# Real GDP growth rates and healthcare spending – comparison between the G7 and the EM7 countries

**DOI:** 10.1186/s12992-020-00590-3

**Published:** 2020-07-16

**Authors:** Mihajlo Jakovljevic, Yuriy Timofeyev, Chhabi Lal Ranabhat, Paula Odete Fernandes, João Paulo Teixeira, Nemanja Rancic, Vladimir Reshetnikov

**Affiliations:** 1 Department of Global Health Economics and Policy, Faculty of Medical Sciences, University of Kragujevac, Kragujevac, Serbia; 2 Institute of Comparative Economic Studies, Hosei University Tokyo, Tokyo, Japan; 3 N.A.Semashko Department of Public Health and Healthcare, I.M. Sechenov First Moscow State Medical University (Sechenov University), Moscow, Russia; 4 National Research University Higher School of Economics, Moscow, Russia; 5 Policy Research Institute, Kathmandu, Nepal; 6 Manmohan Memorial Institute of Health Science, Kathmandu, Nepal; 7 UNIAG, Instituto Politécnico de Bragança, Bragança, Portugal; 8 CEDRI and UNIAG, Instituto Politécnico de Bragança, Bragança, Portugal; 9 Faculty of Medicine of the Military Medical Academy, University of Defence, Belgrade, Serbia

**Keywords:** Real GDP growth, Healthcare expenditure, G7, EM7, Out-of-pocket expenditure

## Abstract

**Background:**

Accelerated globalisation has substantially contributed to the rise of emerging markets worldwide. The G7 and Emerging Markets Seven (EM7) behaved in significantly different macroeconomic ways before, during, and after the 2008 Global Crisis. Average real GDP growth rates remained substantially higher among the EM7, while unemployment rates changed their patterns after the crisis. Since 2017, however, approximately one half of the worldwide economic growth is attributable to the EM7, and only a quarter to the G7. This paper aims to analyse the association between the health spending and real GDP growth in the G7 and the EM7 countries.

**Results:**

In terms of GDP growth, the EM7 exhibited a higher degree of resilience during the 2008 crisis, compared to the G7. Unemployment in the G7 nations was rising significantly, compared to pre-recession levels, but, in the EM7, it remained traditionally high. In the G7, the austerity (measured as a percentage of GDP) significantly decreased the public health expenditure, even more so than in the EM7. Out-of-pocket health expenditure grew at a far more concerning pace in the EM7 compared to the G7 during the crisis, exposing the vulnerability of households living close to the poverty line. Regression analysis demonstrated that, in the G7, real GDP growth had a positive impact on out-of-pocket expenditure, measured as a percentage of current health expenditure, expressed as a percentage of GDP (CHE). In the EM7, it negatively affected CHE, CHE per capita, and out-of-pocket expenditure per capita.

**Conclusion:**

The EM7 countries demonstrated stronger endurance, withstanding the consequences of the crisis as compared to the G7 economies. Evidence of this was most visible in real growth and unemployment rates, before, during and after the crisis. It influenced health spending patterns in both groups, although they tended to diverge instead of converge in several important areas.

## Introduction

Accelerated globalisation, taking place primarily after the end of the Cold War Era, has substantially contributed to creating conditions for the occurrence of rapidly developing large economies worldwide, labelled “emerging markets” [1]. World economy growth was generally quite stable from the beginning of the XXI century until the occurrence of the Global Financial Crisis triggered by the Lehman Brothers’ bankruptcy in the USA in autumn 2007. It had disastrous consequences both in the mature, high-income free market economies and in the rapidly growing major emerging economies. In this study, we observe the largest and most representative national economies in both groups. Therefore, the authors of this paper decided to elaborate further on the World Bank’s adopted comparison of real GDP growth rates and healthcare spending between the first group presented by the G7 nations and the second one marked as Emerging Markets Seven (EM7). The G7 became a formal group in 1976, before the oil crisis. The EM7 was observed as a group of similar economies by John Hawksworth and Gordon Cookson at PricewaterhouseCoopers in 2006, 30 years later [2].

These two groups behaved in significantly different macroeconomic ways before, during, and after the crisis. Average real GDP growth rates remained substantially higher among the EM7, while unemployment rates changed the pre-crisis pattern into a novel post-crisis one. Worldwide economic growth began to accelerate again in 2017 and continued in 2018–2019. However, approximately one half of this growth is attributable to the EM7 and only one quarter to the G7 nations. This gradual, but profound change in global health expenditure is largely attributable to financial globalisation and we seek to understand the underlying causality. Medical technology dissemination, from the rich industrialised Global North to the Global South, took place alongside manufacturing capacities migration, mostly over the past half century. Alongside increased living standards and rapid urbanisation, these changes led to an increased demand for medical goods and services, coupled with a strengthened supply, by both local providers and global multinationals. Big Pharma is a typical example, since innovative pharmaceuticals comprise a much higher share of health spending in low- and middle income countries and Asia, in comparison to the West. All of these gradual, but profound, evolutionary developments contributed to the growing purchasing power of both citizens and governments throughout the Third World nations, which led to their steadily growing share of global health spending. The purpose of this paper is to analyse the association between the health spending and real GDP growth in the G7 and the EM7 countries.

Observing the 2000–2016 period is particularly useful to understand the ability of these emerging nations to invest into healthcare [3–7]. So far, the existing studies focused on certain countries only [8, 9]. There is a large body of seminal evidence focusing on understanding the mutual relationship between the overall pace of economic development and the healthcare attributable spending [10]. From a historical perspective, it was earlier discovered that periods of abundant prosperity created a wealth surplus that was largely allocated towards increasing human longevity and combat diseases [11]. Much later, the feedback loop between the population health and nation’s economic productivity was discovered [12]. Thus, the original perception of medical care, as a purely consumptive branch [13] of the economy, was replaced with the investment concept [14]. Higher health spending led to increased healthy life expectancy [15] and cost savings in the long run [16]. Today, a variety of contemporary health economic literature focuses on discovering the explanatory variables between the real Gross Domestic Product (GDP) growth and the health spending dynamics among modern societies [17]. We seek to understand underlying hidden patterns and long-term trends in increasing national wealth allocated for medical care provision. Since the early 1990s, insufficient health expenditure has led to unacceptably high, out-of-pocket spending by ordinary citizens, gradually increasing, in terms of percentage of GDP [18].

### Data and methods

For the analysis, we used data provided by the Brookings Institute and the World Bank. The G7 included the USA, Japan, Germany, the UK, France, Italy, Canada, while the Emerging Seven Markets (EM7) encompassed China, India, Russia, Brazil, Indonesia, Mexico, and Turkey. The time period analysed was 2000–2016, although some data, within this period, was incomplete. Another data source used was the WHO Global Health Expenditure Database for the following indicators of health expenditure: Current health expenditure (CHE) as percentage of GDP, current health expenditure (CHE) per capita in USD constant 2010, current health expenditure (CHE) per capita in PPP (constant 2011 international USD), domestic general government health expenditure (GGHE-D) as percentage of current health expenditure (CHE), out-of-pocket Expenditure (OOPS) per capita in PPP International USD, out-of-pocket (OOPS) as percentage of current health expenditure (CHE).

The International Monetary Fund (IMF) served as a further, complimentary data source for the following variables: GDP (nationwide in terms of million PPP) and real GDP growth rate (percentage).

We utilised panel regression analysis with country- and year fixed effects, separately, for the G7 and the EM7 countries. We specified the basic model as follows:

$$ {\boldsymbol{Y}}_{it}={\beta}_1\ast Real\  GDP\ {Growth}_{it}+{\boldsymbol{\beta} \boldsymbol{X}}_{it}+{\alpha}_i+{t}_t+{\varepsilon}_{it}, $$

where *α*_*i*_ is an intercept for each country; *t*_*t*_ is an intercept for each year; ***Y***_*it*_ was a vector of dependent variables (*i* is country, *t* is year), such as current health expenditure (CHE) as percentage of GDP (models 1–2), CHE per capita in US$ constant 2010 (models 3–4), CHE per capita in PPP in constant 2011 international USD (models 5–6), domestic general government health expenditure (GGHE-D) as percentage of CHE (models 7–8), out-of-pocket expenditure (OOPS) per capita in PPP international USD (models 9–10), OOPS as percentage of CHE (models 11–12); *ε*_*it*_ is the error term. Real GDP growth was an independent variable, while ***X***_***it***_ was a vector of control variables, which included GDP per capita, inflation, unemployment, population density, and life expectancy. The last indicator was included as a proxy for the burden of all diseases [19]. In the extended model, we included governance effectiveness and the number of physicians per 1000 people. *β*_*1*_ is the coefficient for the independent variable, while ***β*** is a vector of the coefficients for the control variables. Regression analyses were performed using STATA software, version 14.

## Results

### Patterns analysis based on group’s averages

**Fig. 1:**
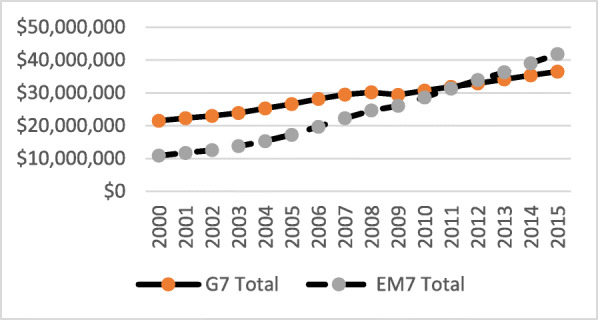
Gross Domestic Product in million current PPP (constant 2011 international USD) in the G7 and the EM7 in 2000–2015. *Source*: IMF

**Fig. 2:**
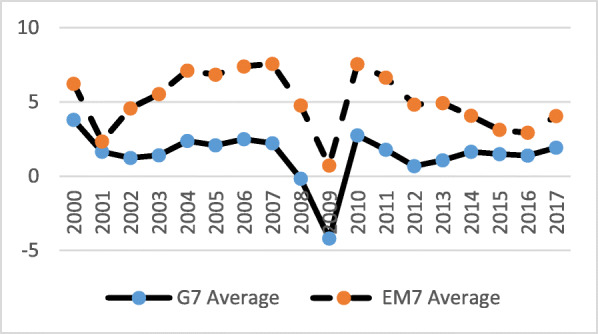
Real GDP growth (Annual percent change) in the G7 and the EM7 in 2000–2017. *Source*: IMF

**Fig. 3:**
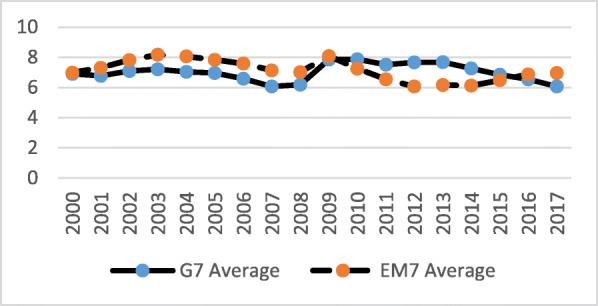
Unemployment rate (Percent) in the G7 and the EM7 in 2000–2017. *Source*: IMF

Over the 16 years (2000–2015), total GDP for both groups of nations grew significantly, while the EM7 grew at a much faster pace (Fig. 1). In early 2012, the EM7 GDP exceeded the G7’s. Both groups reacted to the global macroeconomic crisis in a similar way and economic activity and recovery went mostly in a diminishing, parallel manner. Before, during and after the crisis, the average real GDP growth rates of the EM7 economies were almost constantly twice as high as those of the G7 (Fig. 2). Unemployment rates were, on average, slightly lower among the G7 before the crisis (2000–2008) and somewhat higher among the G7 after the crisis (2009–2016) (Fig. 3).

These dynamic changes affected health spending in a non-linear and unpredictable way. We identified the following patterns.

**Fig. 4:**
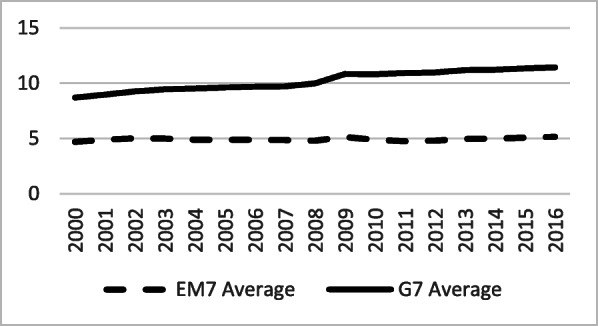
Current Health Expenditure (CHE) as percentage of GDP in the G7 and the EM7 in 2000–2016. *Source*: WHO Global Health Expenditure Database

**Fig. 5:**
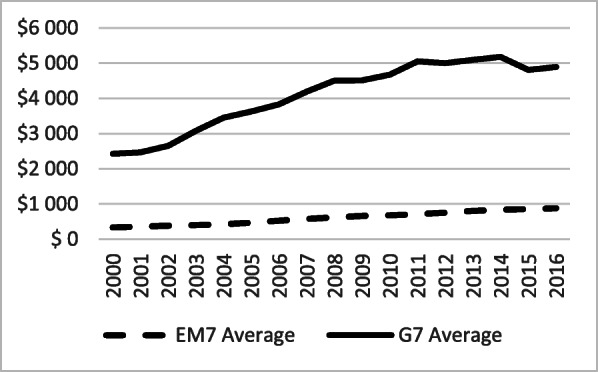
Current Health Expenditure (CHE) per Capita in PPP (current international USD) in the G7 and the EM7 in 2000–2016. Source: WHO Global Health Expenditure Database

Current health expenditure (CHE, expressed as a percentage of GDP) was steadily growing in both groups (Fig. 4). However, it remained significantly higher among the G7 during the entire period. The average difference of 3.85 percentage points between the two groups, in 2000, increased to almost 6 percentage points in 2015. This is in clear contrast with the overall stronger GDP growth trend in the EM7, pointing at a lower priority of health investment among governments in emerging economies and a shifting of newly acquired national wealth towards other priority areas. In per capita PPP terms, upward trends are obvious, but strongly divergent in favour of the G7, following the changes noted above (Fig. 5).

**Fig. 6:**
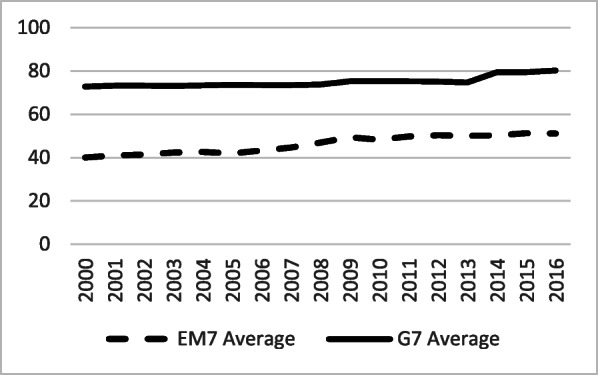
Domestic General Government Health Expenditure (GGHE-D) as a percentage Current Health Expenditure (CHE) in the G7 and the EM7 in 2000–2016. *Source*: WHO Global Health Expenditure Database

Domestic governmental health expenditure, expressed as a percentage of CHE, also exhibited a distinctive difference (Fig. 6). The G7 level was exceptionally stable at 72–74% with, essentially, no change at the group average level over the entire period. At the same time, the EM7 governmental share of spending rose from 35% in 2000 to 50% in 2015. There was a strong upward growth in the early 2000s and a sudden drop due to some governments’ austerity measures in reaction to the 2010 global crisis. Recovery to pre-crisis levels was already evident in 2011.

**Fig. 7:**
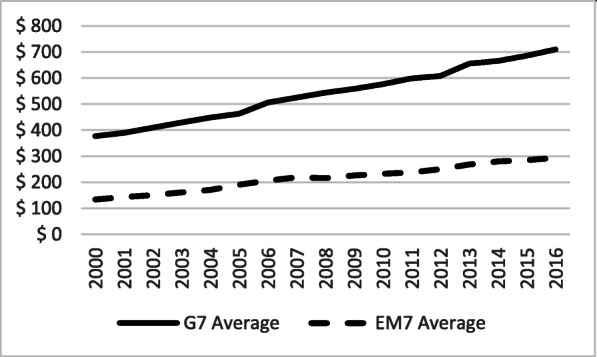
Out-of-Pocket Expenditure (OOPS) per Capita in PPP International USD in the G7 and the EM7 in 2000–2016. *Source*: WHO Global Health Expenditure Database

**Fig. 8:**
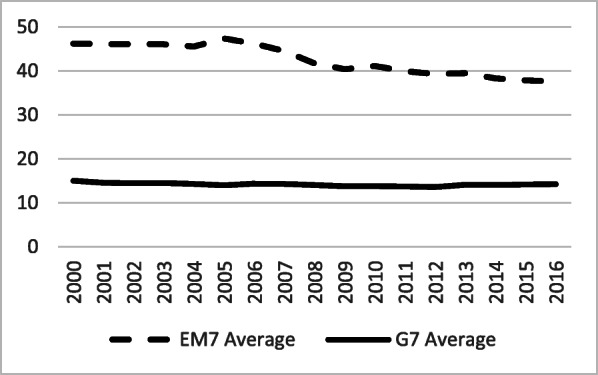
Out-of-Pocket (OOPS) as a percentage of Current Health Expenditure (CHE) in the G7 and the EM7 in 2000–2016, *Source*: WHO Global Health Expenditure Database

Out-of-pocket health expenditure, expressed in USD PPP basis per capita, had more than doubled among the EM7, from $135 on average, in 2000, up to $298 in 2015 (Fig. 7). Among the G7, this increase was also substantial, jumping from $377 in 2000, to $666 in 2015. Out-of-pocket spending, observed as a percentage share of GDP, was surprisingly quite stable among the G7 (reducing from 15 to 14%), while it was downsizing among the EM7 nations from 46 to 39% (Fig. 8).

### Panel regression analysis

Interestingly, real GDP growth affects different aspects of healthcare expenditure in the compared groups. In the G7 countries, real GDP growth had a positive, statistically and economically significant impact on out-of-pocket expenditure, expressed as a percentage of CHE only (see Table 1). In the EM7 countries, real GDP growth had a negative, statistically and economically significant effect on current health expenditure, expressed as a percentage of GDP, CHE per capita, in PPP in constant 2011 international USD, and out-of-pocket expenditure (OOPS) per capita in PPP international USD (see Table 2). The impact of other control variables was not systematic in both groups. Real GDP growth had a negative, statistically and economically significant effect only on current health expenditure, expressed as a percentage of GDP and CHE per capita in USD constant 2010. Tables 3 and 4 provide descriptive statistics and cross-correlation matrices for the variables for the G7 and the EM7 countries respectively. Tables 1 and 2 provide models estimates for the G7 and the EM7 countries respectively. Variance inflation factors from preliminary OLS regressions do not suggest issues of multicollinearity.

**Table 1:** Panel regression analysis estimates with fixed effects for the G7

	(1)	(2)	(3)	(4)	(5)	(6)	(7)	(8)	(9)	(10)	(11)	(12)
VARIABLES	chegdp	chegdp	chepc	chepc	chepcppp	chepcppp	gghed	gghed	oopspcppp	oopspcppp	oopsche	oopsche
Real GDP growth	−.0507*	−.0484	− 38.97	4.62	− 12.78	− 1.920	−.0834	.477	2.094	3.987	.164**	.229**
	(.0276)	(.0433)	(24.97)	(36.66)	(12.32)	(19.65)	(.228)	(.432)	(1.879)	(3.219)	(.0650)	(.0971)
GDP per capita	.000155***	.000170***	.202***	.225***	.231***	.255***	.00168***	.00198***	.0154***	.0169***	−.000178***	−7.25e-05
	(2.81e-05)	(3.49e-05)	(.0254)	(.0295)	(.0125)	(.0158)	(.000232)	(.000348)	(.00191)	(.00259)	(6.61e-05)	(7.82e-05)
Inflation	.0321	.0344	43.79	92.47	−23.41	− 2.185	−.873*	−.689	−9.396**	−4.997	−.245*	−.0956
	(.0603)	(.0696)	(54.61)	(59.00)	(26.94)	(31.63)	(.498)	(.695)	(4.109)	(5.181)	(.142)	(.156)
Unemployment	.134***	.146***	87.39**	87.91*	113.0***	125.3***	.227	.182	.876	−.602	−.186*	−.0924
	(.0411)	(.0527)	(37.25)	(44.64)	(18.38)	(23.93)	(.340)	(.526)	(2.803)	(3.920)	(.0969)	(.118)
Life expectancy	−.0665	−.119	− 141.9	−288.0	− 364.9***	−464.8***	−7.235***	−7.824***	13.35	7.657	.464	−.468
	(.161)	(.206)	(146.1)	(174.7)	(72.08)	(93.63)	(1.331)	(2.058)	(1.99)	(15.34)	(.380)	(.463)
Population density	.0313*	.0485**	−7.196	−12.09	6.089	8.356	.100	.0132	4.435***	5.285***	.141***	.159***
	(.0173)	(.0203)	(15.71)	(17.18)	(7.751)	(9.207)	(.143)	(.202)	(1.182)	(1.508)	(.0409)	(.0455)
Governance effectiveness		1.095**		− 1473***		131.6		−8.189		47.44		.655
		(.496)		(42.4)		(225.3)		(4.953)		(36.91)		(1.113)
Physicians		−.219		−319.3		−243.9		−3.741		−21.58		1.433*
		(.351)		(297.7)		(159.6)		(3.508)		(26.14)		(.788)
Constant	3.560	3.647	8626	23,070*	22,949***	30,040***	577.3***	645.5***	− 1858**	− 1545	− 39.13	24.11
	(1.77)	(13.94)	(9758)	(11,814)	(4814)	(6333)	(88.93)	(139.2)	(734.2)	(1037)	(25.39)	(31.28)
Observations	119	76	119	76	119	76	119	76	119	76	119	76
R-squared	.749	.774	.811	.842	.942	.949	.431	.496	.907	.899	.239	.349
Number of countries	7	7	7	7	7	7	7	7	7	7	7	7

*CHEGDP* Current Health Expenditure vs Gross Domestic Product (national), *CHEPC* Current Health Expenditure per Capita (nominal currency terms), *CHEPCPPP* Current Health Expenditure per Capita (Purchase Power Parity currency terms), *GGHE-D; OOPSPCPPP* Out of Pocket Spending Current Health Expenditure; Purchase Power Parity currency terms), *OOPSCHE* Out of Pocket Spending Current Health Expenditure

Standard errors in parentheses. *** *p* < 0.01, ** p < 0.05, * *p* < 0.1

**Table 2:** Panel regression analysis estimates with fixed effects for the EM7

	(1)	(2)	(3)	(4)	(5)	(6)	(7)	(8)	(9)	(10)	(11)	(12)
VARIABLES	chegdp	chegdp	chepc	chepc	chepcppp	chepcppp	gghed	gghed	oopspcppp	oopspcppp	oopsche	oopsche
Real GDP growth	−.0472***	−.0402***	−.162	−.374	−7.521***	−6.794***	.0295	.192	−3.343***	−2.845*	.0440	−.0334
	(.0109)	(.0134)	(3.202)	(4.188)	(1.756)	(1.983)	(.164)	(.194)	(1.107)	(1.536)	(.169)	(.191)
GDP per capita	−3.95e-05**	−3.43e-05*	.0169***	.0235***	.0416***	.0467***	.00122***	.00101***	.00900***	.00872***	−.00103***	−.00115***
	(1.73e-05)	(1.93e-05)	(.00508)	(.00602)	(.00279)	(.00285)	(.000261)	(.000279)	(.00176)	(.00221)	(.000268)	(.000275)
Inflation	−.0341**	−.0208	−8.901**	−11.76**	−.613	−2.142	−.167	.0763	1.057	1.792	.0803	−.0299
	(.0145)	(.0145)	(4.271)	(4.531)	(2.343)	(2.146)	(.219)	(.210)	(1.476)	(1.662)	(.225)	(.207)
Unemployment	.0826***	.0962***	−13.70	−1.13	8.963*	6.856	1.722***	2.366***	−6.646**	−3.928	−1.435***	−1.620***
	(.0287)	(.0351)	(8.441)	(1.93)	(4.629)	(5.177)	(.433)	(.507)	(2.917)	(4.009)	(.444)	(.500)
Life expectancy	.152***	.111*	51.86***	43.07**	25.54***	12.08	−.780	−.492	1.83**	16.42**	1.378*	2.143***
	(.0514)	(.0562)	(15.15)	(17.51)	(8.309)	(8.294)	(.778)	(.813)	(5.236)	(6.423)	(.797)	(.801)
Population density	−.00877**	−.00886**	−3.924***	−2.771**	−2.359***	−1.415**	.144**	.0870	−.662	−.981*	−.204***	−.216***
	(.00420)	(.00440)	(1.236)	(1.371)	(.678)	(.649)	(.0635)	(.0636)	(.427)	(.503)	(.0651)	(.0627)
Governance effectiveness		.0923		−245.1***		−87.88**		5.885		−53.18		−1.655
		(.295)		(91.83)		(43.49)		(4.260)		(33.68)		(4.199)
Physicians		.113		61.08		36.39**		4.134**		−21.28		−5.664***
		(.123)		(38.41)		(18.19)		(1.782)		(14.09)		(1.756)
Constant	−4.454	−1.788	− 2947***	− 2631**	− 1415***	−662.0	6.28	36.71	−526.0	−856.6**	−1.45	−49.19
	(3.182)	(3.511)	(937.4)	(1094)	(514.1)	(518.1)	(48.13)	(5.76)	(324.0)	(401.2)	(49.34)	(5.03)
Observations	119	87	119	87	119	87	119	87	119	87	119	87
R-squared	.279	.289	.697	.739	.946	.961	.466	.582	.782	.786	.339	.505
Number of countries	7	7	7	7	7	7	7	7	7	7	7	7

*CHEGDP* Current Health Expenditure vs Gross Domestic Product (national), *CHEPC* Current Health Expenditure per Capita (nominal currency terms), *CHEPCPPP* Current Health Expenditure per Capita (Purchase Power Parity currency terms), *GGHE-D; OOPSPCPPP* Out of Pocket Spending Current Health Expenditure; Purchase Power Parity currency terms), *OOPSCHE* Out of Pocket Spending Current Health Expenditure

Standard errors in parentheses. *** *p* < 0.01, ** *p* < 0.05, * p < 0.1

**Table 3:** Summary statistics and cross-correlation matrix for the G7

Variable	Obs	Mean	Std. Dev.	Min	Max	chegdp	chepc	chepcppp	gghed	oopspcppp	oopsche	rgdpgr	gdppc	inf	unemp	lifeexp	popdens	goveff	phys
CHEGDP	119	10.214	2.492	5.972	17.073	1													
CHEPC	119	4086.352	1724.677	1520.454	9869.742	.9153*	1												
CHEPCPPP	119	3917.370	1715.360	1570.207	9869.742	.9558*	.9656*	1											
GGHE-D	119	74.939	11.018	44.189	85.138	−.6475*	−.4937*	−.5418*	1										
OOPSPCPPP	119	538.452	22.296	182.132	1094.227	.7720*	.7764*	.8375*	−.6037*	1									
OOPSCHE	119	14.174	4.163	7.138	26.464	−.2852*	−.2946*	−.2374*	−.0739	.3125*	1								
Real GDP growth	119	1.398	1.931	−5.600	5.200	.0145	.0119	.0298	−.1189	−.0421	−.1284	1							
GDP per capita	119	37,026.510	6972.833	26,293.750	57,807.660	.7865*	.9033*	.9166*	−.3426*	.8131*	−.1675	.0088	1						
Inflation	119	1.433	1.038	−1.381	3.856	.1288	.1126	.1199	−.3161*	.1285	−.0157	.2013*	.1189	1					
Unemployment	119	7.051	2.166	3.100	12.683	.0100	−.1400	−.0858	−.0613	−.0093	.1768	−.1822*	−.1691	.1101	1				
Life expectancy	119	8.432	1.724	76.637	83.985	−.3329*	−.1979*	−.2470*	.5391*	−.1120	.2512*	−.2863*	−.0137	−.3953*	.0470	1			
Population density	119	17.634	116.480	3.374	351.339	−.5283*	−.4402*	−.4862*	.6143*	−.4314*	.1015	−.1871*	−.3890*	−.4170*	−.2660*	.4128*	1		
Gov. effectiveness	112	1.457	.433	.198	1.990	.2031*	.2129*	.1862*	−.0725	−.1823	−.7186*	.3435*	.1806	.0531	−.3939*	−.3437*	−.2335*	1	
Physicians	81	2.949	.758	1.887	4.400	−.1461	−.2109	−.1461	.2468*	.0508	.4547*	−.2744*	−.0779	.0622	.4660*	.2096	.2488*	−.6026*	1

*CHEGDP* Current Health Expenditure vs Gross Domestic Product (national), *CHEPC* Current Health Expenditure per Capita (nominal currency terms), *CHEPCPPP* Current Health Expenditure per Capita (Purchase Power Parity currency terms), *GGHE-D; OOPSPCPPP* Out of Pocket Spending Current Health Expenditure; Purchase Power Parity currency terms), *OOPSCHE* Out of Pocket Spending Current Health Expenditure

* *p* < 0.05

**Table 4:** Summary statistics and cross-correlation matrix for the EM7

Variable	Obs	Mean	Std. Dev.	Min	Max	chegdp	chepc	chepcppp	gghed	oopspcppp	oopsche	rgdpgr	gdppc	inf	unemp	lifeexp	popdens	goveff	phys
CHEGDP	119	4.915	1.677	1.916	9.206	1													
CHEPC	119	32.469	265.220	16.254	1025.490	.7033*	1												
CHEPCPPP	119	605.822	396.038	82.294	1401.007	.7263*	.9411*	1											
GGHE-D	119	46.145	16.832	17.982	8.498	.2552*	.5454*	.6327*	1										
OOPSPCPPP	119	215.886	126.762	39.367	538.159	.6703*	.8061*	.8570*	.3241*	1									
OOPSCHE	119	42.594	15.770	14.508	74.106	−.4273*	−.6118*	−.6808*	−.8923*	−.3086*	1								
Real GDP growth	119	5.129	3.808	−7.800	14.200	−.4219*	−.4594*	−.5317*	−.1989*	−.5464*	.2695*	1							
GDP per capita	119	1156.540	6266.597	2039.533	26,223.770	.3615*	.8006*	.8907*	.7692*	.7242*	−.6924*	−.4121*	1						
Inflation	119	5.731	3.290	−.711	2.374	.0352	.2149*	.3417*	.3464*	.1533	−.3536*	−.3398*	.4218*	1					
Unemployment	119	6.065	2.588	2.268	12.552	.4242*	.3455*	.4518*	.6315*	.0674	−.8202*	−.2318*	.3781*	.3277*	1				
Life expectancy	119	71.074	3.768	62.505	76.860	.4291*	.6057*	.5686*	.4818*	.5104*	−.4673*	−.1821*	.5338*	−.0275	.1734	1			
Population density	119	122.078	124.591	8.716	445.484	−.5247*	−.6054*	−.6699*	−.6040*	−.5871*	.7204*	.3695*	−.6074*	−.0398	−.5818*	−.4659*	1		
Gov.effectiveness	112	−.044	.248	−.720	.409	.1662	.1847	.1412	.2804*	.0881	−.1472	.0213	.1463	−.1147	−.0621	.6674*	.0821	1	
Physicians	93	1.681	1.111	.130	4.309	.3512*	.5362*	.6579*	.5789*	.6061*	−.4651*	−.2447*	.6748*	.2252*	.2406*	.1513	−.6126*	−.1676	1

*CHEGDP* Current Health Expenditure vs Gross Domestic Product (national), *CHEPC* Current Health Expenditure per Capita (nominal currency terms), *CHEPCPPP* Current Health Expenditure per Capita (Purchase Power Parity currency terms), *GGHE-D; OOPSPCPPP* Out of Pocket Spending Current Health Expenditure; Purchase Power Parity currency terms), *OOPSCHE* Out of Pocket Spending Current Health Expenditure

* *p* < .05

In Table 1, columns 1-2 contain the panel regression estimates for current health expenditure in GDP (CHE), for our basic and extended model respectively. Similarly, we represent the results for the remaining models. Regressions 3 and 4 present the results for per capita current health expenditure. Regressions 5 and 6 provide the results for CHE per capita in PPP in constant 2011 international USD. Regressions 7 and 8 provide the results for domestic general government health expenditure (GGHE-D) as a percentage of CHE. Regressions 9 and 10 provide the results for out-of-pocket expenditure (OOPS) per capita in PPP international USD. Finally, regressions 11 and 12 provide the results for OOPS as a percentage of CHE.

In Table 2, columns 1-2 contain the panel regression estimates for current health expenditure in GDP (CHE), for our basic and extended models respectively. Similarly, we represent the results for the remaining models. Regressions 3 and 4 presentthe results for per capita current health expenditure. Regressions 5 and 6 provide the results for CHE per capita in PPP in constant 2011 international USD. Regressions 7 and 8 provide the results for domestic general government health expenditure (GGHE-D) as a percentage of CHE. Regressions 9 and 10 provide the results for out-of-pocket expenditure (OOPS) per capita in PPP international USD. Finally, regressions 11 and 12 provide the results for OOPS as a percentage of CHE.

In the EM7 countries, real GDP growth had a negative, statistically and economically significant effect on current health expenditure expressed as a percentage of GDP, CHE per capita in PPP in constant 2011 international USD, and out-of-pocket expenditure (OOPS) per capita in PPP international USD (see Table 2). The impact of other control variables was not systematic in both groups. Real GDP growth had a negative, statistically and economically significant effect only on current health expenditure expressed as a percentage of GDP and CHE per capita in USD constant 2010.

Regarding the effects of control variables, there is a statistically and economically significant negative effect of governance effectiveness on per capita current health expenditure and CHE per capita in PPP in constant 2011 international USD. There is a statistically and economically significant impact of the number of physicians on CHE per capita in PPP in constant 2011 international USD (positive) and domestic general government health expenditure (GGHE-D) as a percentage of CHE (positive), and out-of-pocket expenditure as a percentage of CHE (negative).

## Discussion

The world economy has experienced profound changes in the past few decades and is expected to undergo uneven globalisation trends [20]. The increasing influence of the large emerging markets on the global economy has become an important issue that calls for more research and policy attention [21]. This growth is expected to be primarily driven by the emerging markets and developing nations, with the EM7 economies growing at an annual average rate of almost 3.5% during the next 34 years [22]. In contrast, an annual average growth rate of 1.6% is predicted for the G7 countries [23]. It further elucidates that the EM7 could account for nearly 50% of the globe’s GDP by 2050, while the G7’s share of global GDP might decline to just over 20%. It is due to population ageing [24] and reduction of natural resources [25]. It clearly indicates that the EM7 is expanding, while the G7 is shrinking [26].

However, it takes more time to implement universal healthcare (UHC) in the EM7 [27] because there is still no policy and program priority for health [28]. Except for the United States, the G7 countries have already achieved UHC [29]. Coverage of universal health insurance should be a high priority for the EM7 countries. Ordinary citizens’ capacity concerning the acquisition of UHC’s benefits spread efficiently, but not fast enough to follow-up disproportionate rapid growth of out-of-pocket spending [30]. In the XXI century, the G7 countries have invested in health around 10% of GDP each year [31], along with similar amounts allocated to infrastructure [15]. In contrast, the EM7 countries have invested in health less than 5 % GDP [32] and set priority to infrastructure and economic growth.

Lastly, the results of the regression analysis, related to the composition of indicators affected by the real GDP growth, as well as the predicted direction of the effect can be attributed to the differences in budget planning priorities, and GDP per capita, in the compared groups.

A set of economic inefficiencies among emerging markets are also attributable to the double economic burden of the unliquidated pool of infectious diseases associated with non-communicable infections [33]. The contribution of population ageing to the growing demand for long-term medical care and pharmaceuticals is probably the most vivid, when comparing Japan [34] with China [35]. These two are the most representative of both groups. In demographic terms, Japan remains in the most advanced stage of population ageing [36]. Yet, even with the recent abolishment of one-child policies [37], China has exhibited only a sudden, but temporary upward shift in fertility rates during the early 2010s, returning to the ratio of 1.1 child per woman [38]. This is far below the simple population replacement threshold of 2.1, and China is most likely to become the fastest ageing nation by 2060 [39].

In addition to the accumulation of incidence of autoimmune [40], cancer [41], and dementia [42] morbidity among elderly citizens, here we face another important phenomenon. It is the so-called “the last year of life” [43]. Its costs of intensive, palliative, and home-based medical care, requiring nursing staff, due to the gradual disappearance of family caregiving across Asia [44], generates costs that are, on average, equal to the entire life-time medical consumption of that individual citizen.

Thus, the economic implications for the health system workload and the financial burden for the social support systems, in the societies with ever-larger populations of elderly citizens, are clear in both the G7 and the EM7. Yet, the latter group of countries finds itself confronted with a much more serious challenge. Namely, population ageing, as the third demographic transition, does not only become a global phenomenon (with 17–18 outlier African nations [45, 46] and Afghanistan [47]), but it is accelerating across the globe. Unlike the Western hemisphere [48], in the EM7, such changes were driven by the spreading of globalisation and sexual revolution during and after the XX century [49], leading to the absorption of women into the labour markets worldwide [50]. The ultimate outcome of this equation was the creation of financial incentives by contemporary societies, Eastern and Western alike, to attract women into the workforce, which, in turn, had a negative effect on the child-per-woman ratio [51]. Here, we come up to the important disadvantage of the emerging nations in this struggle [52]. Ageing, itself, in Western European societies, had its roots in the XIX century [53]. Thus, if one observes the time that was necessary to double the population of senior citizens, from 7 to 14%, in France, it took 115 years, while in Brazil, it took 21 years only. It is clear that the G7 nations, experiencing these changes, historically, in a much earlier momentum, had far more time to evolve, gradually, their social support and health insurance coverage systems to meet the challenge [54]. However, in the emerging, rapidly developing nations, which, with the notable exception of the former Soviet Union [55], were largely non-industrialised countries, it happened much faster.

Indonesia [56], Mexico [57], and Turkey [58] face similar problems related to the double burden of infectious illnesses, non-communicable diseases, and lack of social capital and medical staff capacities to cover these needs. These same matters were already elaborated on in academic literature on BRICS [59], with Russia having historically the earliest developed capacities in preventative medicine and screening procedures [60]. Even today, in a leading Chinese nation, with abundant national welfare [61], cancer-screening procedures, leading to serious long-term savings, are only being systematically pursued in coastal and some urban areas [62].

The approaching health policy reforms in the EM7 is likely to enforce these priorities, even despite the reluctance of their governments to push for higher healthcare investments [63]. Actually, among the BRICS, as the core subgroup of the EM7, all states except India, have managed to increase their GDP share of national health spending from one to two percentage points, on average, since 1990 [64]. This positive trend needs more financial resources and development to be on a par with the G7 countries [65].

This is how we try to explain the core finding of this research. How could it be possible that the EM7, despite their better performance in terms of real GDP growth throughout the crisis, did not channel more welfare into population health? The answer lies in the fact that the XIX century European-style health systems were entirely built upon in the era of sustainable demographic growth [66]. Working citizens contributed to various social and health insurance models, through mandatory taxes, to support the devaluating and insufficiently large pensions and retirement funds for the elderly [67]. Long decades of the post-WWII European social evolution resulted in the creation of the welfare state [68]. In France, an average citizen could enjoy up to 24 years of state-sponsored pension with full pension and release from any mandatory work after his or her retirement [69]. Given the current fertility rates and the increased longevity of post-war generations of baby boomers, these retirements have long since disappeared [70]. We face, nowadays, ever-shrinking cohorts of work-capable citizens at their best age. At the same time, cohorts of the elderly become, annually, evermore increasing in number, leading to a severe distortion of healthy demographic pyramids [71]. The only partial exemption to this case, driven by Latin American immigration, is the USA [72]. All other Western nations follow the very same pattern, with Germany and Italy being among the worst ones, in the most advanced stage of the third demographic transition [73]. Here, we easily observe that the base of taxpayers is getting smaller and the base of tax consumers is getting larger and heavier [74]. Currently foreseen strategies, some of them imposed by the European Commission, refer to extending life work age [75] and thresholds for retirements including re-integration of cancer-survivors [76] and other people, with decreased working ability, back to the labour markets. These measures, including immigration of foreign-born workforces, so far imply the limited outreach [77]. Therefore, health expenditure among the G7 is likely to continue growing, at least twice as fast as the overall economy (with the notable exception of Japan) in the upcoming decades [78]. Obviously, mature economies of the G7 and rapidly evolving economies of the EM7, each following its own distinctive historical pathway, are likely to conform to similar challenges, but from entirely different perspectives [79]. Likewise, their strategies to cope with the burden of medical care spending, and citizen out-of-pocket spending, in particular, are likely to be profoundly different and tailored to their own needs [80].

## Conclusion

Globalisation and its underlying processes has contributed to the creation of conditions for the rise of the EM7 economies. Major emerging markets exhibited a stronger endurance, to withstand the consequences of the global economic crisis, in comparison with traditional, industrial high-income nations [81]. Evidence of this is most visible in the real growth rates and unemployment rate levels, before, during and after the crisis. This situation affected health spending patterns in both groups, although they tend to diverge, instead of converge in several important areas.

It was quite surprising to discover that GDP was growing faster than out-of-pocket health spending. One should not forget that, in absolute terms, it is substantially higher, today, compared to the beginning of the XXI century in both groups of nations. Keeping in mind social insurance programs and the affordability of medical care among the G7 citizens, it is well known that such change will lead to far worse consequences among the emerging nations. The vulnerability of ordinary citizens, particularly those living in rural areas, far away from the cities and industrial areas, might lead to increased health expenditure.

Regression analysis demonstrated that real GDP growth affects different aspects of healthcare expenditure in the compared groups. In the G7 countries, real GDP growth had a positive impact on out-of-pocket expenditure, expressed as a percentage of CHE only. In the EM7 countries, it affected, negatively, the current health expenditure, expressed as a percentage of GDP, CHE per capita in PPP in constant 2011 international USD, and out-of-pocket expenditure per capita in PPP international USD.

Governments in the emerging economies shall be challenged to increase their investment into healthcare systems, in order to support current economy growth in the long run [82]. Currently, serious social inequalities, in terms of access and affordability of medical care and pharmaceuticals, among most of the EM7 countries, shall be smoothed to some extent by the continuous growth of the middle class in China and India and economic recovery in Russia and Brazil in 2015–2016. The G7 nations preserve their stable allocation of resources for healthcare in the first decades of the XXI century, although with concerning long-term sustainability perspective, primarily due to population ageing consequences [83–86].

## Data Availability

The datasets generated and/or analysed during the current study are available in the WHO Global Health Expenditure Database, https://apps.who.int/nha/database; the World Development Indicators Database, https://databank.worldbank.org/source/world-development-indicators; and the IMF Data Mapper repository, http://www.imf.org/external/datamapper/

## References

[1] Sliburyte L, Ostaseviciute R (2009). Theoretical aspects of economic globalization impacts on emerging economies. Econ Manag.

[2] PricewaterhouseCoopers. The World in 2050. https://www.pwc.com/gx/en/world-2050/assets/pwc-world-in-2050-summary-report-feb-2017.pdf. Accessed 12 Nov 2019.

[3] Huidrom R, Kose A, Ohnsorge FL (2017). How important are spillovers from major emerging markets? Policy research working paper; no. WPS 8093.

[4] Kose A (2017). Big emerging market economies versus the G7: Which group will drive the upswing in global growth?.

[5] Morgan D, Astolfi R (2015). Financial impact of the GFC: health care spending across the OECD. Health Econ Policy Law.

[6] Jakovljevic M, Getzen TE (2016). Growth of global health spending share in low and middle income countries. Front Pharmacol.

[7] Jakovljevic M, Potapchik E, Popovich L, Barik D, Getzen TE (2017). Evolving health expenditure landscape of the BRICS nations and projections to 2025. Health Econ.

[8] Baji P, Péntek M, Boncz I, Brodszky V, Loblova O, Brodszky N (2015). The impact of the recession on health care expenditure - how does the Czech Republic, Hungary, Poland and Slovakia compare to other OECD countries?. Soc Econ.

[9] Maresso A, Mladovsky P, Thomson S, Sagan A, Karanikolos M, Richardson E, Cylus J, Evetovits T, Jowett M, Figueras J, Kluge H (2015). Economic crisis, health systems and health in Europe: Country experience.

[10] Smith S, Newhouse JP, Freeland MS (2009). Income, insurance, and technology: why does health spending outpace economic growth?. Health Aff.

[11] Lindert PH. Growing Public. In: Social spending and economic growth since the eighteenth century, vol. 1. New York: Cambridge University Press; 2004.

[12] Loeppke R, Nicholson S, Taitel M, Sweeney M, Haufle V, Kessler RC (2008). The impact of an integrated population health enhancement and disease management program on employee health risk, health conditions, and productivity. Popul Health Manag.

[13] Jakovljevic M, Ogura S. Health economics at the crossroads of centuries–from the past to the future. Front Public Health. 2016. 10.3389/fpubh.2016.00115. 10.3389/fpubh.2016.00115 PMC4899886 27376055

[14] Rivera B, Currais L (2003). The effect of health investment on growth: a causality analysis. Int Adv Econ Res.

[15] Ranabhat CL, Atkinson J, Park MB, Kim CB, Jakovljevic M (2018). The influence of universal health coverage on life expectancy at birth (LEAB) and healthy life expectancy (HALE): a multi-country cross-sectional study. Front Pharmacol.

[16] Jakovljevic MB (2013). Resource allocation strategies in southeastern European health policy. Eur J Health Econ.

[17] Getzen TE, Poullier JP (1992). International health spending forecasts: concepts and evaluation. Soc Sci Med.

[18] Alam K, Mahal A (2014). Economic impacts of health shocks on households in low and middle income countries: a review of the literature. Glob Health.

[19] Murray CJ, Lopez AD (2013). Measuring the global burden of disease. N Engl J Med.

[20] Pieterse JN. Multipolar globalization: Emerging economies and development: Routledge; 2017. https://www.routledge.com/Multipolar-Globalization-Emerging-Economies-and-Development/Pieterse/p/book/9781138232280.

[21] Khuong VM. Sources of growth in the world economy: a comparison of G7 and E7 economies. Cambridge: Academic Press; 2019.

[22] Jakovljevic M, Matter-Walstra K, Sugahara T (2020). Cost-effectiveness and resource allocation (CERA) 18 years of evolution: maturity of adulthood and promise beyond tomorrow. Cost Eff Resour Alloc.

[23] Global Security Review (2019). Emerging Economies Will Hold Increasing Amounts of Global Economic Power by 2050.

[24] Jakovljevic MB, Netz Y, Buttigieg SC, Adany R, Laaser U, Varjacic M (2018). Population aging and migration – history and UN forecasts in the EU-28 and its east and south near neighbourhood – one century perspective 1950-2050. Global Health.

[25] Gordon RJ. The rise and fall of American growth: the us standard of living since the civil war, vol. 70. Princeton: Princeton University Press; 2017.

[26] Wade RH (2011). Emerging world order? From multipolarity to multilateralism in the G20, the World Bank, and the IMF. Polit Soc.

[27] Ranabhat CL, Jakovljevic M, Dhimal M, Kim CB (2019). Structural factors responsible for universal health coverage in low-and middle-income countries: results from 118 countries. Front Public Health.

[28] Marten R, McIntyre D, Travassos C, Shishkin S, Longde W, Reddy S, Vega J (2014). An assessment of progress towards universal health coverage in Brazil, Russia, India, China, and South Africa (BRICS). Lancet.

[29] Tull K (2019). G7 Universal Health Coverage (UHC).

[30] Blumenthal D, Hsiao W (2005). Privatization and its discontents – The evolving Chinese health care system. Mass Med Soc.

[31] World Bank (2019). Current health expenditure (CHE).

[32] Jakovljevic MB (2016). Comparison of historical medical spending patterns among the BRICS and G7. J Med Econ.

[33] Jakovljevic M, Jakab M, Gerdtham U, McDaid D, Ogura S, Varavikova E, Merrick J, Adany R, Okunade A, Getzen TE (2019). Comparative financing analysis and political economy of noncommunicable diseases. J Med Econ.

[34] Ogura S, Jakovljevic M (2018). Global population aging – health care, social and economic consequences. Front Public Health.

[35] Sun Q, Lu N, Jiang N, Lou VW. Intention to use respite services among informal care-givers of frail older adults in China: the role of care needs change. Ageing Soc. 2020:1–20. 10.1017/S0144686X20000628.

[36] Hong PK, Goh LG, Sharipova D. Ageing in Asia: beyond the Astana declaration towards financing long-term Care for all; comment on “financing long-term care: lessons from Japan”. Int J Health Policy Manag. 2020. 10.34172/ijhpm.2020.15. 10.34172/ijhpm.2020.15 PMC7947709 32610781

[37] Zhuang Y, Jiang Y, Li B. Fertility intention and related factors in China: findings from the 2017 National Fertility Survey. China Popul Dev Stud. 2020. 10.1007/s42379-020-00053-7.

[38] Hui X, Peng W, Liao L, Dai N, Zhao F. Can quantitative easing population policy rescue china’s sinking fertility rate? A comparative examination between Jiangsu and Zhejiang. Eur J Geograp. 2020. 10.4000/cybergeo.34066.

[39] Li Q, Reuser M, Kraus C, Alho J (2009). Ageing of a giant: a stochastic population forecast for China, 2006–2060. J Popul Res.

[40] Watad A, Bragazzi NL, Adawi M, Amital H, Toubi E, Porat BS, Shoenfeld Y (2017). Autoimmunity in the elderly: insights from basic science and clinics-a mini-review. Gerontology.

[41] Jakovljevic M, Folic M, Dagovic A (2015). Hospital oncology costs among the cohort of elderly in an aging south-eastern European nation. Farmeconomia Health Econ Ther Pathways.

[42] Prince M, Ali GC, Guerchet M, Prina AM, Albanese E, Wu YT (2016). Recent global trends in the prevalence and incidence of dementia, and survival with dementia. Alzheimers Res Ther.

[43] Zhu B, Li F, Wang C (2018). Tracking hospital costs in the last year of life — the Shanghai experience. Biosci Trends.

[44] Hinton L, Tran D, Nguyen TN, Ho J, Gitlin L. Interventions to support family caregivers of people living with dementia in high, middle and low-income countries in Asia: a scoping review. BMJ Glob Health. 2019;4(6). 10.1136/bmjgh-2019-001830. 10.1136/bmjgh-2019-001830 PMC6861057 31798992

[45] Bloom DE, Canning D, Fink G (2007). Realizing the demographic dividend: Is Africa any different?.

[46] Kirk D, Pillet B (1998). Fertility levels, trends, and differentials in sub-Saharan Africa in the 1980s and 1990s. Stud Fam Plan.

[47] Gaan N (2015). Youth bulge: constraining and reshaping transition to Liberal democracy in Afghanistan. India Quart.

[48] Silies EM (2015). Taking the pill after the ‘sexual revolution’: female contraceptive decisions in England and West Germany in the 1970s. Eur Rev History.

[49] Benería L, Berik G, Floro M. Gender, development and globalization: economics as if all people mattered. New York: Routledge; 2015.

[50] Tétreault MA. Women and revolution in Africa, Asia, and the new world. Columbia: Univ of South Carolina Press; 1994.

[51] Caucutt EM, Guner N, Knowles J (2002). Why do women wait? Matching, wage inequality, and the incentives for fertility delay. Rev Econ Dyn.

[52] Jakovljevic MB, Vukovic M, Fontanesi J (2016). Life expectancy and health expenditure evolution in Eastern Europe — DiD and DEA analysis. Exp Rev Pharmacoecon Outcomes Res.

[53] Caldwell JC (2006). The Western fertility decline: reflections from a chronological perspective. J Popul Res.

[54] Dieleman JL, Campbell M, Chapin A, Eldrenkamp E, Fan VY, Haakenstad A (2017). A. Future and potential spending on health 2015–40: development assistance for health, and government, prepaid private, and out-of-pocket health spending in 184 countries. Lancet.

[55] Gertler PJ, Molyneaux JW (1994). How economic development and family planning programs combined to reduce Indonesian fertility. Demography.

[56] McCAA R. Women's position, family and fertility decline in Parral (Mexico) 1777-1930. Annales de démographie historique. 1989;233–43. 10.3406/adh.1989.1743. 10.3406/adh.1989.1743 11622367

[57] Yavuz S (2008). Fertility decline in Turkey from the 1980s onwards: patterns by main language groups.

[58] Misra R (2015). Impact of demographic dividend on economic growth: a study of BRICS and the EU. Int Stud.

[59] Arsentyev EV, Reshetnikov VA (2018). To the biography of NA Semashko: on the work of the first People’s commissar of health in 1920–1925. History Med.

[60] Huang X (2015). Four worlds of welfare: understanding subnational variation in Chinese social health insurance. China Q.

[61] Guo LW, Zhang SK, Liu SZ (2020). Analysis of endoscopic screening compliance and related factors among high risk population of upper gastrointestinal cancer in urban areas of Henan Province from 2013 to 2017. Zhonghua Yu Fang Yi Xue Za Zhi.

[62] Jakovljevic M, Groot W, Souliotis K. Health care financing and affordability in the emerging global markets. Front Public Health. 2016. 10.3389/fpubh.2016.00002. 10.3389/fpubh.2016.00002 PMC4720748 26835444

[63] Jakovljevic MB (2015). BRIC’s growing share of global health spending and their diverging pathways. Front Public Health.

[64] Dieleman J, Campbell M, Chapin A (2017). Evolution and patterns of global health financing 1995–2014: development assistance for health, and government, prepaid private, and out-of-pocket health spending in 184 countries. Lancet.

[65] Bloom DE, Canning D, Sevilla J. Economic Growth and the Demographic Transition (No. w8685). Cambridge: National Bureau of Economic Research; 2001.

[66] Bazzana D. Ageing population and pension system sustainability: reforms and redistributive implications. Economia Politica. 2020:1–22. 10.1007/s40888-020-00183-8.

[67] Flora P, Alber J. Modernization, democratization, and the development of welfare states in Western Europe. In: Development of Welfare states in Europe and America. New York: Routledge; 2017. p. 37–80.

[68] Hénin PY, Weitzenblum T (2005). Welfare effects of alternative pension reforms: assessing the transition costs for French socio-occupational groups. J Pension Econ Finance.

[69] Roberts K (2012). The end of the long baby-boomer generation. J Youth Stud.

[70] Sekicki-Pavlenko OO (2016). The aging process of the population in modern Russia and its consequences. Demografija.

[71] Davis C (1983). US Hispanics: changing the face of America. Popul Bullet.

[72] Grant J, Hoorens S, Sivadasan S, Loo MVH, Davanzo J, Hale L, Butz W (2006). (2006). Trends in European fertility: should Europe try to increase its fertility rate or just manage the consequences?. Int J Androl.

[73] Ogura S, Kadoda T (2010). Effects of public policies and labor market on the fertility of Japanese women: analyses of municipal data. Fertil Pub Policy.

[74] European Cooperation in Science and Technology (2019). Gender and Work Age Extension in Europe.

[75] de Boer AG (2014). The European Cancer and work network: CANWON. J Occup Rehabil.

[76] Prskawetz A, Fent T, Guest R (2008). Workforce aging and labor productivity: the role of supply and demand for labor in the G7 countries. Popul Dev Rev.

[77] Hall RE, Jones CI (2007). The value of life and the rise in health spending. Quart J Econ.

[78] Khanna T, Palepu KG, Sinha J (2005). Strategies that fit emerging markets. Harv Bus Rev.

[79] Rancic N, Jakovljevic M (2016). Long term health spending alongside population aging in N-11 emerging nations. East Eur Bus Econ J.

[80] Husain AM, Mody A, Rogoff KS (2005). Exchange rate regime durability and performance in developing versus advanced economies. J Monet Econ.

[81] Jakovljevic M, Timofeyev Y, Ekkert NV, Fedorova JV, Skvirskaya G, Bolevich S, Reshetnikov VA (2019). The impact of health expenditures on public health in BRICS nations. J Sport Health Sci.

[82] Williams G, Cylus J, Roubal T, Ong P, Barber S. Sustainable health financing with an ageing population: will population ageing lead to uncontrolled health expenditure growth? World Health Organization; 2019. 31820890

[83] Gunnarsson V, Carcillo S, Verhoeven M. Education and health in G7 countries: achieving better outcomes with less spending (no. 7-263). Washington, D.C.: International Monetary Fund; 2007.

[84] Nili F, Hitiris T. Health Care Expenditure and Cost Containment in the G7 Countries. https://core.ac.uk/download/pdf/6755941.pdf. Accessed 25 May 2020.

[85] Ogura S, Jakovljevic M (2014). Health financing constrained by population aging-an opportunity to learn from Japanese experience. Serbian J Exp Clin Res.

[86] Reshetnikov V, Arsentyev E, Boljevic S, Timofeyev Y, Jakovljevic M (2019). Analysis of the financing of Russian health care over the past 100 years. Int J Environ Res Public Health.

